# TOR Signaling Pathway in Cardiac Aging and Heart Failure

**DOI:** 10.3390/biom11020168

**Published:** 2021-01-27

**Authors:** Nastaran Daneshgar, Peter S. Rabinovitch, Dao-Fu Dai

**Affiliations:** 1 Department of Pathology, University of Iowa, Iowa City, IA 52242, USA; nastaran-daneshgar@uiowa.edu; 2 Department of Pathology, University of Washington, Seattle, WA 98195, USA; peterr@medicine.washington.edu

**Keywords:** mTOR, aging, cardiac aging, heart failure, rapamycin, caloric restriction

## Abstract

Mechanistic Target of Rapamycin (mTOR) signaling is a key regulator of cellular metabolism, integrating nutrient sensing with cell growth. Over the past two decades, studies on the mTOR pathway have revealed that mTOR complex 1 controls life span, health span, and aging by modulating key cellular processes such as protein synthesis, autophagy, and mitochondrial function, mainly through its downstream substrates. Thus, the mTOR pathway regulates both physiological and pathological processes in the heart from embryonic cardiovascular development to maintenance of cardiac homeostasis in postnatal life. In this regard, the dysregulation of mTOR signaling has been linked to many age-related pathologies, including heart failure and age-related cardiac dysfunction. In this review, we highlight recent advances of the impact of mTOR complex 1 pathway and its regulators on aging and, more specifically, cardiac aging and heart failure.

## 1. Introduction

Aging is characterized by progressive decline in physiological functions and functional reserve of several organ systems, promoting the vulnerability to disease and death.

Cardiovascular diseases (CVDs) are the leading cause of death worldwide [1], with elderly individuals (aged > 70 years) comprising approximately two-thirds of those dying from CVD. Given the exponential increases in the prevalence of several cardiovascular diseases with aging, old age is recognized as one of the strongest and independent risk factors for CVD [2]. With the aging of the American and worldwide population, the prevalence of CVD increases over time and imposes a huge economic burden due to increased healthcare spending. For example, the prevalence of heart failure in the US increased from 5.7 million adults in 2009 to 6.2 million in 2016 [1], approximately a 10% increase in less than 10 years. Therefore, it is critical to understand the molecular mechanisms of cardiac aging and how aging predisposes to heart failure, in order to develop interventions to target these mechanisms. This review will focus on the role of mammalian target of rapamycin (mTOR) signaling in cardiac aging and heart failure, including the signaling network and potential treatments targeting the mTOR complex 1 pathway in aging and heart failure (Table 1).

## 2. Cardiac Aging in Human and Animal Models

According to the data from Framingham Heart Study (FHS) and Baltimore Longitudinal Studies on Aging (BLSA), cardiovascular aging in apparently healthy adults manifests as left ventricular (LV) hypertrophy, diastolic dysfunction, diminished LV systolic reserve capacity, increased arterial stiffness, and impaired endothelial function in cardiovascular tissues [40,41]. Age is a risk factor, which is independent of other extrinsic cardiovascular risk factors such as hypertension, smoking, diabetes, obesity, hyperlipidemia, etc. The pathophysiology of cardiovascular aging includes maladaptation of cellular metabolism, cardiomyocyte dysfunction, decrease in angiogenesis, and increase in tissue scarring (fibrosis) in aged myocardial tissues [42,43].

Valvular changes related to aging mainly comprise valvular sclerosis, defined as myxomatous degeneration and collagen deposition. Approximately 30–80% of elderly individuals suffer from aortic valve sclerosis [44]. Aortic sclerosis progression to aortic stenosis (AS), characterized by increased leaflet calcification and decreased leaflet mobility, is an indicator of increased CVD risk [45]. In addition to AS, the prevalence of aortic regurgitation is also high among the elderly population, approximately 13–16% [46], which results in left ventricular dilation and dysfunction over time due to chronic volume overload. Another valvulopathy associated with aging is mitral annular calcification (MAC), which commonly accompanies aortic valve sclerosis [47]. Studies have shown that subjects with MAC are at increased risk of heart failure, atrial fibrillation, coronary and vascular diseases, and major adverse cardiovascular events [48]. With advancing age, pathologies such as thickening of the arterial wall and degeneration of the elastic and collagen in the media lead to dilation, elongation, and stiffness of the wall of the aorta [49]. In addition, age-related aortic unfolding (elongation and widening of the aortic arch) is related to increased aortic stiffness [50]. All in all, cardiac ventricular and valvular alterations due to aging are detrimental to cardiac functional reserve capacity and facilitate the progression of heart failure, leaving the aged heart susceptible to stress.

Aging is accompanied by declining diastolic function, as determined by the ratio of early to late ventricular filling (E/A) by Doppler echocardiography. The maximal exercise capacity decreases with age, while systolic function, measured by ejection fraction, is preserved at rest. Aging also leads to increase in left ventricular (LV) wall thickness, which indicates increased LV hypertrophy [40,41]. The participants of the BLSA study were apparently healthy, without hypertension, or clinically apparent cardiovascular diseases; thus, all of the above changes are likely manifestations of cardiac aging itself.

Aging is associated with the impairment of LV early diastolic filling (E wave by Doppler imaging), which is likely due to reduced ventricular compliance because of LV hypertrophy and increased interstitial fibrosis, along with slower reuptake of cytosolic calcium in myocardial cells by sarcoplasmic reticulum, which further delays relaxation. The decrease in early diastolic filling demands atrial contraction during the late diastolic phase (A wave by Doppler imaging) [51] to contribute to a larger fraction of LV filling. This leads to increased atrial pressure and subsequent atrial hypertrophy, which predisposes to the development of atrial fibrillation, the prevalence of which also increases with age. Diastolic dysfunction promotes exercise intolerance in the elderly population and may also lead to the symptoms of heart failure, despite preserved ejection fraction, defined as heart failure with preserved ejection fraction (HFpEF). HFpEF is prevalent in older individuals and markedly increases the risk of mortality [52]. More than 50% of patients over the age of 75 with the clinical diagnosis of congestive heart failure suffer from HFpEF, and many of them are clinically unrecognized and untreated [52].

Murine cardiac aging phenotypes closely resemble human cardiac aging, characterized by left ventricular hypertrophy and decreased diastolic function [40,53]. Since many strains of mice do not develop spontaneous hypertension or diabetes with age, the observed aging changes are likely attributable to intrinsic aging. These make the mouse model a good model to study intrinsic cardiac aging. Our previous studies showed age-dependent cardiac pathology in aged mice that include an increase in left ventricular mass index (LVMI) and left atrial dimension, increased diastolic dysfunction, impaired myocardial performance index (predominantly due to impaired diastolic function), enlarged left atrium due to increased LV end-diastolic pressure, and also increase in myocardial fibrosis [54]. Systolic function was only modestly affected, similar to that observed in apparently healthy adults in the FHS and BLSA studies discussed above. The histopathology of old murine hearts includes cardiomyocyte hypertrophy (increased myocardial fiber size); increased variation in myocyte fiber size, cytoplasmic vacuolization, and hyalinization; collapse of sarcomeres; increased interstitial fibrosis, mineralization, arteriosclerosis, and arteriolosclerosis; and increased deposition of collagen and senile amyloid. Increased cardiomyocyte apoptosis has also been observed in some studies [55]. All of these changes are collectively designated as age-associated cardiomyopathy.

## 3. TOR Signaling in Aging

**Figure 1:**
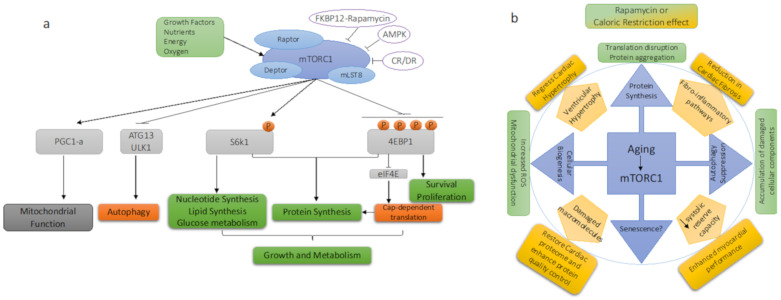
(**a**) mTORC1 signaling; (**b**) mTORC1 signaling in aging and cardiac aging modulated by Rapamycin and/or Caloric restriction.

The mammalian target of rapamycin (mTOR) signaling pathway senses a wide variety of environmental signals, including nutrients, amino acids, hormones, and mitogens, transducing adaptive responses within the cell (Figure 1a). These responses may include metabolic adaptation by regulating transcription and translation, autophagy, apoptosis, mitochondrial biogenesis, lipid metabolism, glycolysis, and inflammation. mTOR is a serine/threonine kinase in the PI3K family, forming two distinct complexes—mTOR complex 1 (mTORC1) and complex 2 (mTORC2) [56]. The mTORC1 complex consists of three major components: mTOR itself; Raptor (regulatory protein associated with mTOR), which facilitates recruitment of substrate and localization of the complex; and mLST8 (mammalian lethal with Sec13 protein 8, also known as GβL) [57,58,59].

mTORC1 has two distinct mechanisms to control protein synthesis. First, it promotes mRNA translation through its downstream substrates and effectors, S6K1 and eukaryotic initiation factor 4E (eIF4E) binding proteins (4E-BP) [60]. Second, it drives protein synthesis through the regulation of ribosome biogenesis [61,62]. This is accomplished by regulating the transcription of genes coding for ribosomal proteins [63], ribosome biogenesis factors [64], and tRNAs [65,66]. The activation of mTORC1 upregulates the expression of genes related to growth and metabolism [56] and downregulates genes involved in cellular stress adaptation [67]. One major downstream signal, S6K1, when phosphorylated and activated by mTORC1, itself phosphorylates programmed cell death protein 4 (PDCD4) and destines it for degradation [68]. The role of PDCD4 is to impede protein translation by inhibiting eIF4A helicase, its inhibition ultimately leading to accelerated ribosome biogenesis. Ribosomal protein S6, the substrate of S6K1, has been widely used as a marker of mTORC1 activity [62]. The ability of mTORC1 to phosphorylate and activate S6K1 is controlled by Raptor [69] and is also sensitive to ATP alterations [70]. S6K1 exerts its regulatory effect on cell size and growth and metabolism [71] through the activity of adenosine monophosphate (AMP)-activated protein kinase (AMPK) [72]. This links the two major nutrient and energy sensor signaling pathways in the cells: mTOR and AMPK.

In addition to S6K1, mTORC1 also phosphorylates its other target, 4E-BP1, releasing it from eIF4E and de-repressing cap-dependent initiation of translation [10]. The eukaryotic initiation factor 4F (eIF4F) complex regulates the mRNA cap-binding protein eIF4E and scaffold protein eIF4G association, thereby mediating growth-dependent protein synthesis, which in turn leads to circularization of mRNAs and an increased translation rate [73].

The mTOR pathway is one of the most important pathways involved in the aging process. The implication of mTOR signaling in the process of aging was first shown in the *C. elegans* model system by the observation that decreased expression of mTOR homologs (ceTOR, formerly *let-363*) or Raptor (*daf-15*) extended worm life span [74,75]. Subsequently, extended longevity related to reduction in TOR signaling was shown in budding yeast [3], *Drosophila* [14], and mice [76,77]. Although the exact mechanism of how mTOR signaling affects aging is not fully understood, several studies suggest that mTORC1 inhibition delays aging by decreasing mRNA translation mainly through 4E-BP1, increasing proteostasis and decreasing oxidative stress [56]. This is supported by the observation of extended longevity in mice with loss of the mTORC1 substrate S6 kinase (S6K1) [29]. The deletion of S6K1 in mice led to increased life span; resistance to age-related pathologies, including dysfunctions in bone, motor, and immune systems; and insulin resistance and also prompted gene expression patterns resembling those seen in caloric restriction (CR) or AMPK activation [29]. The fact that S6K1 is regulated by nutrient and hormonal signals suggests its critical role underlying the beneficial effect of CR. Besides S6K1, the 4EBP1 pathway has also been implicated in aging. The downregulation of various components of the eIF4F cap-binding complex extends life span in *C. elegans* [78].

The third major downstream pathway affected by the activation of mTORC1 is the inhibitory effect on autophagy (Figure 1a). Autophagy is a homeostatic process to maintain healthy macromolecules within cells through the degradation and turnover of damaged or defective cellular components, in order to protect cells against stress. Proteostasis, including autophagic function, declines with age, in parallel with the development of several degenerative diseases, including cardiac aging. Impaired autophagy causes a decline in quality control mechanisms, leading to accumulation of damaged organelles and other cellular components, which subsequently manifest as degenerative changes seen in several organ systems. Activation of mTORC1 promotes cellular growth, and in this context the inhibition of recycling of cellular components via autophagy makes sense. mTORC1 inhibits autophagy through two key effectors, unc-51-like autophagy-activating kinase 1 (ULK1, homologous to ATG1 in yeast) and ATG13 [79]. To form autophagosomes, ULK1 and ATG13 bind FAK family kinase-interacting protein (FIP200) and ATG101 [80]. The decline of autophagic capacity in many aged tissues can be restored by mTOR inhibition [81]. This activation of autophagy by mTOR inhibition assists in the clearance of damaged organelles and harmful macromolecules, thus restricting propagation of harmful causes of age-dependent degeneration. Finally, activation of mTORC1 also regulates membrane trafficking [82] to facilitate the delivery and maturation of several nutrient transporters to the cell surface.

### Caloric Restriction as the Most Reproducible Intervention to Delay Aging via mTORC1 Inhibition and Activation of Autophagy

Caloric restriction, described as decrease in nutrient intake without incurring malnutrition, is the most reproducible intervention shown to extend life span in a broad variety of organisms, ranging from yeast, worm, *Drosophila*, and mice [83]. CR also ameliorates several age-related diseases, including age-related malignancies [84], cardiovascular aging, neurodegenerative and other degenerative diseases such as macular degeneration [85], and age-dependent sensorineural hearing loss [86]. Furthermore, CR has been shown to improve health span in primates [11]. Moderate CR decreases the age-related increase and delayed the onset of age-related pathologies such as diabetes, malignancies, cardiovascular disease, and neurodegeneration [11]. In contrast, caloric intake above an optimal level shortens life span in experimental models. In fact, in flies adding a single essential amino acid, methionine, to dietary restriction is sufficient to abrogate the increase in life span [87], implying the importance of protein synthesis restriction in anti-aging.

Convincing evidence has shown that CR exerts anti-aging effects primarily through inhibition of the mTORC1 pathway, as mTORC1 has a critical role in nutrients and insulin sensing [56]. This is further supported by the absence of additional life span extension by CR in yeast, worms, or flies with decreased mTORC1 signaling [3,14,88]. A study on the downstream signaling of mTORC1 has shown that translational modulation through 4E-BP mediates many of the beneficial effect of dietary restriction (DR) [10]. In *Drosophila*, DR resulted in differential loading of mRNA onto ribosomes compared to flies fed with rich nutrient conditions. DR-enhanced mRNA translation of various mitochondrial gene, especially mitochondrial electron transport components through upregulation of 4E-BP. This led to enhanced mitochondrial activity and extension of *Drosophila* life span, all of which were dependent on 4E-BP [10]. This was further reinforced by the finding that muscle-specific overexpression of 4E-BP in *Drosophila* increased life span and improved proteostasis [89]. Although there is no direct evidence of S6K1 mediating the beneficial effect of CR, as was shown for 4E-BP in *Drosophila* models, the report of life span and health span extension in S6K1 knockout mice and the gene expression patterns resembling the CR effect [29] indicate a critical role of S6K1 in longevity.

Both CR and inhibition of mTORC1 by rapamycin have been shown to enhance autophagic clearance of damaged molecules, improving proteostasis and thereby increasing protein quality control [90,91,92]. A study in mice showed that activation of autophagy through disruption of beclin1 (homologous to atg6 in yeast and flies) can significantly increase life span and health span and diminish age-related cardiac pathologies such as fibrosis [93]. Similarly, suppression of aging phenotypes through activation of autophagy has been shown in yeast, flies, worms, and human cells [94]. Mechanistically, TOR negatively suppresses starvation-induced autophagy by involvement of ATG1. The fact that inhibition of mTOR in ATG-deficient *C. elegans* did not demonstrate life span extension suggests that the pro-longevity effect of mTOR inhibition is at least partly mediated by an autophagy-dependent mechanism [81,95].

## 4. CR and mTORC1 Inhibition in Cardiac Aging

Cardiac aging is associated with impairment in several nutrient and metabolic pathways in the heart, which may directly cause or indirectly affect the functional and structural deterioration of the heart [96,97]. One of the most important pathways is mTORC1, which, as described above, has been shown to be a negative regulator of life span. However, the growth-promoting effects of mTORC1 activation are required for normal cardiac development, cellular growth and maintenance, as well as preservation of cardiac structure and vascular integrity during embryonic and postnatal states [98]. Thus, germline deletion of mTORC1 components in mice is embryonic lethal due to multiple cardiac and vascular abnormalities [99]. The complex interaction of mTOR signaling and cardiac aging is schematically shown in Figure 1b.

*Drosophila* is a good model to study molecular mechanism of cardiac aging, as it has an accessible heart-analog and has significant advantages to other models, such as shorter life span, lower genetic redundancy, and easy manipulation of gene expression. Several genes were altered in *Drosophila* cardiac aging, including genes involved in extracellular matrix remodeling, mitochondrial metabolism, protein handling, and contractile function, findings resembling those of aged mammalian hearts [100]. As one major downstream signal, phosphorylation of 4EBP by mTORC1 activation releases the sequestration of eIF4E (4EBP is an eIF4E binding protein), exposing the phosphorylation sites of eIF4E, which has been shown to be indispensable for *Drosophila* growth through increased protein translation [101]. The overexpression of eIF4E in mammalian cells gives rise to fibroblast transformation and increase in cell size, which is reversed by increased expression of 4E-BP [102]. In *Drosophila*, upregulation of EIF4E alone recapitulated increased TOR effects on insulin signaling and cardiac aging, whereas overexpression of 4EBP in *Drosophila* was shown to protect against cardiac functional aging and promotion of cardiac stress resistance and maintenance of a normal heart rate [6].

Long-term CR attenuates many cardiac aging phenotypes, including cardiac hypertrophy, diastolic dysfunction [103,104,105], as well as age-associated cardiomyopathy in rodents and monkeys [11,106]. In rhesus monkeys, cardiovascular disease is a prevalent age-associated disorder, as it is in humans. The most common cardiovascular diseases found at necropsy of rhesus monkeys include myxomatous valvular degeneration (particularly mitral regurgitation), cardiomyopathy, and myocardial fibrosis. The prevalence of these pathologies was decreased by 50% in the animals subjected to long-term CR [11].

Short-term CR for 10 weeks initiated at middle age (26-month-old) in mice was shown to rejuvenate the aging heart [16]. The findings included the regression of age-dependent cardiac hypertrophy and significant improvement of diastolic function. Interestingly, while there was no significant change in the rate of turnover (half-life) of the cardiac global proteome with age (including measurement of 823 cardiac proteins), the proteome half-lives of old hearts significantly increased by ~30% after short-term CR. This was accompanied by attenuation of age-dependent protein oxidative damage and ubiquitination. Quantitative cardiac proteomics and pathway analysis of proteome changes revealed an age-dependent decreased abundance of proteins involved in mitochondrial function, electron transport chain, citric acid cycle, and fatty acid metabolism as well as increased abundance of proteins involved in glycolysis and oxidative stress response [16]. This age-dependent cardiac proteome remodeling was significantly reversed by short-term CR. Taken together, late-life CR prolongs health span by restoring youthful proteome and reversing alterations in metabolic pathways that are due to aging [107], leading to rejuvenation of multiple organs, including the beneficial effect on cardiac physiology [16]. 

### Rapamycin and Cardiac Aging

Rapamycin (RP), a macrolide from *Streptomyces hygroscopicus* bacteria discovered within the soil of the island of Rapa Nui, was initially described as an anti-fungal drug [108]. However, it is a potent inhibitor of mTOR signaling with anti-tumor and immunosuppression therapeutic applications [56]. As an inhibitor of mTORC1, RP has been shown to prolong not only life span but also health span in mammals and all studied model organisms [5,12,15]. Inhibition of mTORC1 by RP results in overall suppression of protein synthesis, cell growth inhibition, activation of stress response, and autophagy [109]. The inhibition of mTORC1 reduces the energetic burden of translation, decreases oxidative stress, halts the accumulation of deleterious metabolic by-products, and enhances the autophagic removal of damaged macromolecules, leading to enhancement of cellular function [10]. Rapamycin initiated late in life has been shown to extend life span in genetically heterogeneous mice, which is reproducible in multiple different institutions [12]. The life span and health span extension of rapamycin is dose-dependent and affected by sex [110]. RP increased life span more in females than in males at each dose, likely due to the sex difference in RP metabolism and the blood levels achieved by the drug. Although RP has been proposed as a CR mimetic, the relationship between CR and rapamycin in worms and flies is not clearly demonstrated to be epistatic, and indeed, life span in flies is extended by rapamycin at every level of calorie intake [4,5,78,88]. Extensive mammalian metabolomic and transcriptomic studies in blood, liver, and white adipose tissue suggest that rapamycin and DR have distinct, largely non-overlapping effects [111,112,113,114,115]. Several studies have shown that RP-treated mice have distinct endocrine and metabolic alterations compared with mice treated with CR [110]. The discordant metabolic effect of RP and CR is evidenced by distinct alterations of liver proteome after short-term CR or RP treatment [107]. Nevertheless, both CR and RP attenuate protein oxidative damage and improve protein quality. The potential translational values of RP are further supported by studies showing the beneficial effect of transient RP treatment in increasing life span and health span of middle-aged mice, as noted above. The improved health span includes rejuvenation of cardiac aging phenotypes, decrease in non-hematopoietic malignancies, decreased periodontitis, and remodeling of the microbiome [116].

In terms of cardiac aging, the beneficial effects of mTORC1 suppression by RP has been reproducible by multiple laboratories. Aged mice treated with RP for three months improved cardiovascular function and attenuation of cardiac aging pathologies, including heart inflammation and cardiac fibrosis [18]. Studies from our laboratory demonstrated that short-term RP closely resembles the effect of CR in regressing age-dependent cardiac hypertrophy and improvement of diastolic function [16]. This is accompanied by preservation of mitochondrial proteome and improvement of protein quality. Interestingly, RP inhibits ULK phosphorylation and induces autophagy during just the first week of treatment, then returning to baseline at two weeks and after. This observation is concordant with the dynamic changes of mitochondrial biogenesis markers. This transient induction of autophagy and mitochondrial biogenesis suggests that damaged mitochondria are replaced by new mitochondrial biogenesis, hence restoring mitochondrial quality [117].

The potential side effects of RP include immunosuppression and glucose intolerance, which are mainly due to inhibition of mTORC2, while the anti-aging benefits are mostly attributed to mTORC1, rendering the benefits of developing a more specific mTORC1 inhibitors [56]. However, mTORC2 inhibition may be quite detrimental to the heart. Overexpression of heart mTORC2 in flies extends their life span, promotes autophagic flux, and preserves cardiac function with aging [118]. On the other hand, inhibition of liver mTORC2 has been shown to reduce life span in a sex-hormone-dependent manner [119]. Meanwhile, Sestrin, a protein that antagonizes TORC1 while stimulating TORC2, has shown cardioprotective in flies and mice through upregulation of autophagic activities as well as suppressing oxidative stress [120,121].

The detrimental metabolic effects of RP have been documented in several studies, presenting a paradox of improved survival despite metabolic impairment [122]. The unfavorable metabolic effect of RP was observed during the early stages of treatment. As the treatment continued beyond 20 weeks, these effects were diminished and treated mice had increased oxygen consumption and ketogenesis, and markedly enhanced insulin sensitivity [122]. Furthermore, long-term analysis of genetically heterogeneous HET3 mice showed that the effects of rapamycin on glucose homeostasis persist for at least 14 months [123]. The concern of potential side effects of RP, mainly insulin resistance and immunosuppression, has precluded their clinical use as an anti-aging agent. To avoid the side effects, alternative dosing regimens and other RP analogs (Rapalog) [124] have been developed (see Table 1). The application of doses low enough for anti-aging benefits, but avoiding the side effects, or intermittent dosing of RP have been tested in mice. The cardiac benefit of transient RP is sustained even after the cessation of RP treatment, supporting intermittent inhibition of mTORC1 signaling as an effective therapeutic strategy for health span extension [109]. Intermittent RP also provided extended life span without the unwanted glucose intolerance [124,125]. Intriguingly, low doses of mTOR inhibitors have been shown to improve immune function of elderly patients in a small study [126] and to enhance immune function and reduce infections in the elderly in a second study [127]. In addition to intermittent dosing strategies, a number of other dietary and pharmacological strategies are being investigated to safely inhibit mTORC1. These include protein restricted and ketogenic diets [128,129] as well as novel mTORC1-selective rapamycin analogs, which is 40× more selective for mTORC1 than rapamycin and inhibit mTORC1 signaling without impairing glucose homeostasis and substantially reduced or no side effects on lipid metabolism and the immune system [130].

## 5. Rapamycin and the mTORC1 Signaling in Heart Failure

As noted above, aging is a major risk factor for cardiovascular disease, including heart failure. With the rapidly growing aging population, it is estimated that over 8 million people will suffer from heart failure (HF) in the United States by 2030 [131]. This is especially true for HF with preserved ejection fraction (HFpEF), which is more prevalent than HF with reduced ejection fraction (HFrEF) in the elderly population older than 80 years. As discussed above, mTORC1 is a major determinant signaling pathway involved in the regulation of aging and longevity. mTORC1 also serves as a master regulator of several crucial cellular processes, including protein synthesis, cellular growth, proliferation, autophagy, lysosomal function, and cell metabolism. In the heart, mTORC1 is required for embryonic cardiac development and maintenance of cardiac homeostasis in postnatal life. It also plays critical roles in both physiological and pathological processes.

Global disruption of the mTOR gene led to early post-implantation lethality and prohibited embryonic stem cell development [132]. Constitutional cardiac-specific deletion of mTOR at embryonic or early postnatal stage caused dilated cardiomyopathy and early mortality [23] (see Table 1 for summary). These findings emphasize the significance of mTOR for growth and proliferation of both embryonic and early postnatal cardiac development and maintenance [132,133]. Furthermore, mTOR ablation in adult mouse myocardium also led to fatal dilated cardiomyopathy, indicating the role of mTOR in maintaining cardiac physiology and homeostasis. Cardiac mTOR ablation showed accumulation of 4EBP1, indicating that the negative effect of mTOR ablation is mechanistically mediated through 4EBP1 inhibition of protein translation. Interestingly, increase in dephosphorylated 4E-BP1 has also been shown in late-stage HF. Accumulation of 4E-BP1 caused impairment of protein translation and abolish protein synthesis within cardiomyocytes [25].

Conditional homozygous deletion of Raptor (a component of mTORC1) in mouse cardiomyocytes resulted in increased stress, dilated cardiomyopathy, and severe cardiac dilation in response to pressure over overload at 1 week, without adaptive hypertrophy, indicating the essential role of mTORC1 in adaptive ventricular growth and cardiac homeostasis [27]. While mTORC1 is required for adaptive cardiac hypertrophy, excessive mTORC1 activation during chronic stress led to detrimental effects such as increased pathological hypertrophy, accumulation of damaged proteins, and energy stress [134].

As discussed above, mTORC1 inhibition by RP improves cardiac function in both cardiac aging and heart failure. The protective effect of RP against pathological hypertrophy and heart failure has been shown in both the transverse aortic constriction (TAC) model and in hereditary cardiomyopathy [19,135]. Several protective mechanisms have been proposed, including regulation of metabolic pathways and decrease in mTORC1 target proteins like 4EBP1 and S6K1, leading to decreased protein translation. In mouse models with TAC, pressure overload activated phosphorylation of ribosomal S6 protein and eukaryotic translation initiation factor-4E (eIF4E), which were suppressed by RP [17], suggesting a critical role of S6K in mediating cardiac hypertrophy. In contrast, cardiac-specific deletion of both S6K1 and S6K2 in mice showed no significant impact on the progression of physiological (exercise-induced) or pathological (pressure overload) cardiac hypertrophy, indicating that development of cardiac hypertrophy is not critically dependent on S6Ks [30].

To better elucidate the cardioprotective mechanism of mTORC1 inhibition, we tested the role of enhanced 4EBP1, resembling the inhibition of mTORC1 signaling [28], in response to cardiac stresses using multiple mouse models. The first genetic model was a wild-type 4EBP1 overexpression (4EBP1Tg) mouse with an approximately 9-fold increase in cardiac 4EBP1; this demonstrated modest suppression of protein translation. This mouse model had aggravated heart failure in response to pressure overload or concomitant Gαq overexpression. The second genetic model was a mutant 4EBP1 (4EBP1mut, A37/A46), in which the mutant phosphorylation sites are not subject to TOR inhibition (i.e., a constitutively active form of 4EBP1) and a 3× increase in mutant protein. This mouse model demonstrated stronger suppression of protein translation than the 4EBP1Tg model and developed exaggerated heart failure in response to pressure overload (worse than 4EBP1Tg). As expected from the inhibition of protein synthesis, adaptive cardiac hypertrophy was not observed in the 4EBP1mut [28]. To recapitulate the beneficial effect of RP, we applied the third model, a cardiac-specific heterozygous deletion of Raptor, which decreased but not completely abolished mTORC1 activity. Raptor het mice attenuated heart failure in response to pressure overload or Gαq overexpression, and this was accompanied by better preservation of the cardiac proteome, especially the proteins involved in mitochondrial function, glucose metabolism, and the TCA cycle; this is in line with the proteomics pattern of cardiac aging reversal phenotypes [16]. Our study suggested that modest inhibition of mTORC1 (RP or Raptor het) is beneficial, whereas strong suppression of protein translation via enhanced 4EBP1 is detrimental, indicating the necessity of maintaining a level of protein synthesis needed for adaptive cardiac hypertrophy. Taken together, these findings demonstrate that although partial inhibition of mTORC1 by rapamycin [19,135] or raptor has cardioprotective effects, complete ablation of mTORC1 is detrimental [25,27,132,133].

## 6. Conclusions

mTORC1 is required for embryonic development, growth, and maintenance of physiological functions of several organ systems including heart. Pathophysiological functions of mTORC1 pathways in the aging and specifically cardiac aging have been extensively studied. Activation of mTORC1 in response to nutrient signaling promotes protein synthesis, cellular growth, and inhibits autophagy. In contrast, inhibition of mTORC1 by either caloric restriction or rapamycin suppresses overall protein translation and activate autophagy, resulting in enhanced protein quality. In the heart, deletion of mTORC1 components at various life stages is detrimental and lead to dilated cardiomyopathy, whereas modest inhibition of mTORC1 is beneficial in the context of cardiac aging, hypertrophy, and heart failure. Strong suppression of protein translation through activation of 4E-BP1 aggravates heart failure and diminishes adaptive cardiac hypertrophy.

Rapamycin and its related Rapalogs may provide potential therapeutic applications to delay aging and age-related diseases. Remarkable progress has been made in enhancing our understanding of mTOR signaling and its indispensable role in aging and aging-related pathologies.

## Figures and Tables

**Table 1:** Role of mTOR Signaling in Aging and Heart Failure [3,4,5,6,7,8,9,10,11,12,13,14,15,16,17,18,19,20,21,22,23,24,25,26,27,28,29,30,31,32,33,34,35,36,37,38,39].

Interventions	Signaling	Effects on Aging	Cardiac Aging	Heart Failure
Calorie/Dietary Restriction	inhibit mTORC1	Extension of lifespan in yeast [3], worm [4], fruitfly [5]	Drosophila: Protects against functional cardiac aging, maintains normal heart rate and rhythm [6]	Drosophila: Promote cardiac stress resistance
	d4E-BP induction	▪ Protection against cancer, diabetes, atherosclerosis in Drosophila [7,8,9,10], mice ▪ Extension of healthspan in monkey [11]	Mice: protects against cardiomyopathy	N/A
Rapamycin	inhibit mTORC1	▪ Extends lifespan of mice from different strain backgrounds [12,13] ▪ Supresses age-related pathologies in yeast [3], Drosophila [14], worm [15]	▪ Rejuvenates cardiac aging and ameliorate mitochondrial proteome remodeling with aging [16] ▪ Improvement of systolic function [17] ▪ Reduction in cardiac inflammation and fibrosis [18]	Regresses pressure overload induced cardiac hypertrophy [19] and LV fibrosis
mTOR overexpression	Increased mTOR	NA	N/A	▪ Protect against ischemia reperfusion injury and reduce inflammation [20] ▪ Tg of constitutively active mTOR attenuates cardiac injury in OVE26 type 1 diabetic mice [21] ▪ No increase in cardiac mass, preservation of cardiac function and reduced myocardial inflammation [22]
mTOR deletion	Marked suppression of mTORC1	embryonic lethal	NA	Mice: Constitutive cardiac mTOR deletion cause dilated cardiomyopathy and early mortality [23] Mice: Constitutive cardiac mTOR gene deletion at early post-natal stage cause dilated cardiomyopathy and early mortality [24]
				Mice: Cardiac specific inducible deletion in adult led to fatal dilated cardiomyopathy [25]
Raptor deletion	Decreased mTORC1	age related hearing loss [26]	N/A	Mice: Cardiac homozygous deletion: Spontaneous heart failure [27] Mice: Heterozygoug deletion: Ameliorated pressure overload induced HF [28]
S6K1 deletion		Extends murine lifespan, resistance to age related pathologies such as insulin resistance, immune and motor dysfunction [29]	N/A	Mice: No effect on cardiac hypertrophy [30]
				Inhibition of S6K with PF-4708671 protected against MI similar to rapamycin [31]
4EBP1 overexpression	Increased 4EBP1	Drosophila: life span extension, improved proteostasis [10]	Drosophila: Reverses Cardiac functional aging [6]	Aggravates heart failure induced by pressure overload or Gαq overexpression [28]
	Mild suppression of protein translation	Mice: Protects against aging-induced obesity and increase in energy expenditure [32]	N/A	N/A
4EBP1 mutant	Constitutively active 4EBP1 Strong suppression of protein translation	N/A	N/A	Aggravates heart failure, worse than 4EBP1 Tg, adverse remodeling of cardiac proteome, impaired adaptive cardiac hypertrophy due to suppression of protein translation [28]
ULK1 inhibition	Decreased autophagy	Worsen aging-associated diseases	N/A	Mice: Cardiac specific deletion of ULK1 impaired autophagy and caused fissed dysfunctional mitochondria, leading to rapidly progressive cardiomyopathy and early death [33] Mice: Cardiac specific ULK1 knock out had impaired autophagy, increased lipotoxicity and cardiac dysfunction in response to high fat diet [34]
		Promote age-related diseases, including cancer, atherosclerosis, obesity, neurodegeneration and retinopathy [35]		
Rapalogs (everolimus, temsirolimus and deforolimus)		prevents age-related diseases in mice and human, antineoplastic effect [36,37,38]	N/A	Everolimus increased autophagy, decreased remodeling after MI and improved heart function [39]

## Data Availability

Not applicable.
